# 2-(Naphthalen-1-yl)-4-(thio­phen-2-yl­methyl­idene)-1,3-oxazol-5(4*H*)-one

**DOI:** 10.1107/S1600536811016151

**Published:** 2011-05-07

**Authors:** Cevher Gündoğdu, Serap Alp, Yavuz Ergün, Barış Tercan, Tuncer Hökelek

**Affiliations:** a Department of Chemistry, Faculty of Arts and Sciences, Dokuz Eylül University, Tınaztepe, 35160 Buca, Izmir, Turkey; b Department of Physics, Karabük University, 78050, Karabük, Turkey; c Department of Physics, Hacettepe University, 06800 Beytepe, Ankara, Turkey

## Abstract

The asymmetric unit of the title compound, C_18_H_11_NO_2_S, contains two crystallographically independent mol­ecules. In one mol­ecule, the oxazole and thio­phene rings are oriented at dihedral angles of 17.40 (9) and 18.18 (7)° with respect to the naphthalene ring system, while the oxazole and thio­phene rings are oriented to each other at a dihedral angle of 0.86 (9)°. In the other mol­ecule, the corresponding angles are 3.05 (8), 9.62 (6) and 7.02 (8)°, respectively. In each mol­ecule, a weak intra­molecular C—H⋯N hydrogen bond links the oxazole N atom to the naphthalene group. Weak inter­molecular C—H⋯O hydrogen bonding is present in the crystal structure. π–π stacking between the oxazole and thio­phene rings, between the thio­phene and naphthalene rings, and between the oxaozole and naphthalene rings, [centroid–centroid distances = 3.811 (2), 3.889 (2), 3.697 (2) and 3.525 (2) Å] may further stabilize the crystal structure.

## Related literature

For potential applications of the title compound, such as organic light-emitting diodes (OLEDs), organic thin-film transistors (OTFTs), and organic photovoltaics (OPVs) of various aromatic ring-based conjugated polymers, see: Liu *et al.* (2007 ▶); Allard *et al.* (2008 ▶); Woudenbergh *et al.* (2004 ▶); Zhang *et al.* (2007 ▶); Güneş *et al.* (2007 ▶); Soci *et al.* (2007 ▶). For the roles of thio­phene-based mol­ecules widely used in the syntheses of the charge-transporting mol­ecules used in organic field effect transistors, organic solar cells and organic light emitting diodes, see: Mas-Torrent & Rovira (2008 ▶); Shirota & Kageyama (2007 ▶); Varis *et al.* (2006 ▶). For bond-length data, see: Allen *et al.* (1987 ▶).
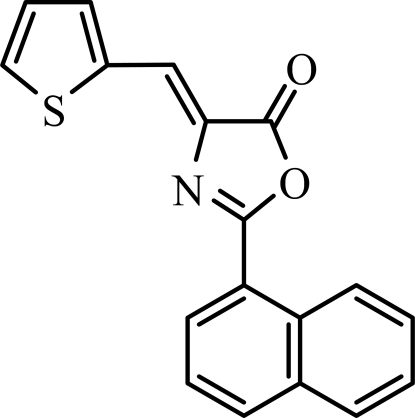


## Experimental

### 

#### Crystal data

C_18_H_11_NO_2_S
*M* *_r_* = 305.35
Monoclinic, 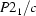

*a* = 11.1509 (3) Å
*b* = 7.0871 (2) Å
*c* = 35.2592 (5) Å
β = 97.914 (4)°
*V* = 2759.91 (12) Å^3^
*Z* = 8
Mo *K*α radiation
μ = 0.24 mm^−1^
*T* = 294 K
0.35 × 0.22 × 0.20 mm

#### Data collection

Bruker Kappa APEXII CCD area-detector diffractometer
Absorption correction: multi-scan (*SADABS*; Bruker, 2005 ▶) *T*_min_ = 0.921, *T*_max_ = 0.953
25128 measured reflections
6899 independent reflections
3925 reflections with *I* > 2σ(*I*)
*R*_int_ = 0.061

#### Refinement

*R*[*F* ^2^ > 2σ(*F* ^2^)] = 0.054
*wR*(*F* ^2^) = 0.133
*S* = 1.01
6899 reflections
405 parameters
2 restraints
H atoms treated by a mixture of independent and constrained refinement
Δρ_max_ = 0.44 e Å^−3^
Δρ_min_ = −0.44 e Å^−3^

### 

Data collection: *APEX2* (Bruker, 2007 ▶); cell refinement: *SAINT* (Bruker, 2007 ▶); data reduction: *SAINT*; program(s) used to solve structure: *SHELXS97* (Sheldrick, 2008 ▶); program(s) used to refine structure: *SHELXL97* (Sheldrick, 2008 ▶); molecular graphics: *ORTEP-3 for Windows* (Farrugia, 1997 ▶); software used to prepare material for publication: *WinGX* (Farrugia, 1999 ▶) and *PLATON* (Spek, 2009 ▶).

## Supplementary Material

Crystal structure: contains datablocks I, global. DOI: 10.1107/S1600536811016151/xu5202sup1.cif

Structure factors: contains datablocks I. DOI: 10.1107/S1600536811016151/xu5202Isup2.hkl

Supplementary material file. DOI: 10.1107/S1600536811016151/xu5202Isup3.cml

Additional supplementary materials:  crystallographic information; 3D view; checkCIF report

## Figures and Tables

**Table 1:** Hydrogen-bond geometry (Å, °)

*D*—H⋯*A*	*D*—H	H⋯*A*	*D*⋯*A*	*D*—H⋯*A*
C3—H3⋯N1	0.93	2.27	2.924 (3)	127
C3′—H3′⋯N1′	0.93	2.29	2.946 (3)	127
C6—H6⋯O2^i^	0.93	2.59	3.483 (3)	160
C9′—H9′⋯O2^ii^	0.93	2.46	3.310 (3)	152
C16′—H16′⋯O2′^iii^	0.93	2.50	3.329 (3)	149

Symmetry codes: (i) 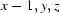; (ii) 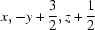; (iii) 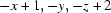.

**Fig. 1.**
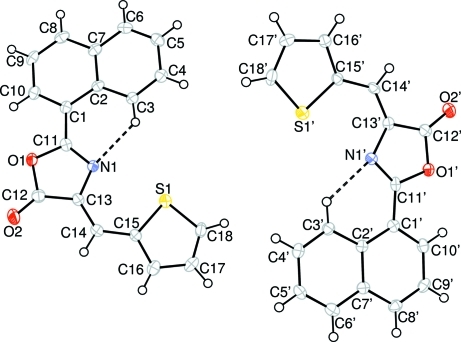
The molecular structure of the title molecule with the atom-numbering scheme. Displacement ellipsoids are drawn at the 50% probability level. The intramolecular C-H···N and C'-H'···N' hydrogen bonds are shown as dashed lines.

**Fig. 2.**
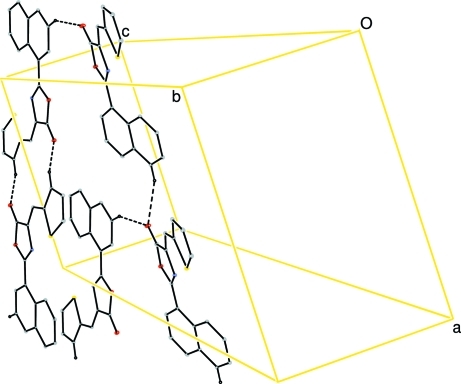
A view of the crystal packing of the title compound. The C-H···O, C'-H'···O and C'-H'···O' hydrogen bonds are shown as dashed lines.

## supplementary crystallographic information

### Comment

The design and syntheses of new conjugated polymers are a significant part of the conducting polymers research and have attracted great attention. Various aromatic ring-based conjugated polymers have been developed for use in potential applications, such as organic light-emitting diodes (OLEDs) (Liu *et al.*, 2007; Allard  *et al.*, 2008), organic thin-film transistors (OTFTs) (Woudenbergh   *et al.*, 2004; Zhang *et al.*, 2007), and organic photovoltaics (OPVs) (Güneş *et al.*, 2007; Soci *et al.*, 2007). Among conducting polymers, polythiophene and its derivatives have become a subject of considerable interest as electrochromic materials, due to their chemical stabilities. Thiophene based molecules are widely used in the sytheses of the charge transporting molecules used in organic field effect transistors, organic solar cells and organic light emitting diodes (Mas-Torrent & Rovira, 2008; Shirota & Kageyama, 2007; Varis *et al.*, 2006). The present study was undertaken to ascertain the crystal structure of the title compound.

The asymmetric unit of the title compound contains two crystallographically independent molecules. Each molecule consists of an oxazol ring, a thiophene ring and a naphthalene group (Fig. 1), where the bond lengths are close to standard values (Allen *et al.*, 1987). In each molecule, the intramolecular C-H···N hydrogen bonds link the oxazol nitrogen atoms to the naphthalene groups (Table 1 and Fig. 1).

An examination of the deviations from the least-squares planes through individual rings shows that rings A (C2—C7), B (C1/C2/C7—C10), C (O1/N1/C11—C13), D (S1/C15—C18) and A' (C2'—C7'), B' (C1'/C2'/C7'—C10'), C' (O1'/N1'/C11'—C13'), D' (S1'/C15'—C18') are planar. The naphthalene groups, containing the rings A, B and A', B' are also nearly planar [with maximum deviations of -0.032 (3) Å for atom C3 and 0.028 (3) Å for atom C4'] with dihedral angles of A/B = 2.28 (8) and A'/B' = 1.65 (8) °. In each molecule, rings C, D and C', D' are oriented with respect to the planar naphthalene groups at dihedral angles of 17.40 (9), 18.18 (7) ° and 3.05 (8), 9.62 (6) °, while the oxazole and thiophene rings are oriented at dihedral angles of 0.86 (9) and 7.02 (8) °,respectively.

In the crystal, intermolecular C'—H'···O' hydrogen bonds link the molecules into centrosymmetric dimers, in which they are also linked through C'-H'···O and C-H···O hydrogen bonds to form a three dimensional network (Table 1 and Fig. 2). The π–π contacts between the oxazol and thiophene rings, between the thiophene and naphthalene rings and between the oxaozole and naphthalene rings Cg3—Cg4^i^, Cg6—Cg8^ii^, Cg6—Cg7^iii^ and Cg5—Cg7^iii^ [symmetry codes: (i) -x, 1/2 + y, 1/2 - z, (ii) -x, 2 - y, -z, (iii) -x, 1 - y, -z, where Cg3, Cg4, Cg5, Cg6, Cg7 and Cg8 are centroids of the rings C (O1/N1/C11—C13), D (S1/C15—C18), A' (C2'—C7'), B' (C1'/C2'/C7'—C10'), C' (O1'/N1'/C11'—C13') and D' (S1'/C15'—C18'), respectively] may further stabilize the structure, with centroid-centroid distances of 3.811 (2), 3.889 (2), 3.697 (2) and 3.525 (2) Å, respectively.

### Experimental

For the preparation of the title compound, (I), thiophene-2-carbaldehyde (0.46 g, 5 mmol), naphthalen-1-yl glycine (1.14 g, 5 mmol), acetic anhydride (2.49 ml, 12 mmol) and sodium acetate (0.41 g, 5 mmol) were heated until the mixture just liquefied, and then heating was continued for a further 2 h at 353 K. After completion of the reaction, ethanol (25 ml) was added and the mixture was kept at room temperature for 18 h. The solid product obtained was purified by washing with cold ethanol, hot water and a small amount of hexane, respectively. It was crystallized from hot ethanol (yield; 0.23 g, 49%, m.p. 460 K).

### Refinement

H14 and H14' atoms are located in a difference Fourier synthesis and refined isotropically. The remaining C-bound H-atoms were positioned geometrically with C—H = 0.93 Å, and constrained to ride on their parent atoms with *U*_iso_(H) = 1.2*U*_eq_(C).

### Crystal data

**Table:**

C_18_H_11_NO_2_S	*F*(000) = 1264
*M**_r_* = 305.35	*D*_x_ = 1.470 Mg m^−^^3^
Monoclinic, *P*2_1_/*c*	Mo *K*α radiation, λ = 0.71073 Å
Hall symbol: -P 2ybc	Cell parameters from 3188 reflections
*a* = 11.1509 (3) Å	θ = 2.3–22.5°
*b* = 7.0871 (2) Å	µ = 0.24 mm^−^^1^
*c* = 35.2592 (5) Å	*T* = 294 K
β = 97.914 (4)°	Block, orange
*V* = 2759.91 (12) Å^3^	0.35 × 0.22 × 0.20 mm
*Z* = 8	

### Data collection

**Table:**

Bruker Kappa APEXII CCD area-detector diffractometer	6899 independent reflections
Radiation source: fine-focus sealed tube	3925 reflections with *I* > 2σ(*I*)
graphite	*R*_int_ = 0.061
φ and ω scans	θ_max_ = 28.4°, θ_min_ = 1.2°
Absorption correction: multi-scan (*SADABS*; Bruker, 2005)	*h* = −14→12
*T*_min_ = 0.921, *T*_max_ = 0.953	*k* = −9→9
25128 measured reflections	*l* = −46→46

### Refinement

**Table:**

Refinement on *F*^2^	Primary atom site location: structure-invariant direct methods
Least-squares matrix: full	Secondary atom site location: difference Fourier map
*R*[*F*^2^ > 2σ(*F*^2^)] = 0.054	Hydrogen site location: inferred from neighbouring sites
*wR*(*F*^2^) = 0.133	H atoms treated by a mixture of independent and constrained refinement
*S* = 1.01	*w* = 1/[σ^2^(*F*_o_^2^) + (0.0482*P*)^2^ + 0.9624*P*] where *P* = (*F*_o_^2^ + 2*F*_c_^2^)/3
6899 reflections	(Δ/σ)_max_ = 0.001
405 parameters	Δρ_max_ = 0.44 e Å^−^^3^
2 restraints	Δρ_min_ = −0.44 e Å^−^^3^

### Special details

**Table:**

Geometry. All esds (except the esd in the dihedral angle between two l.s. planes) are estimated using the full covariance matrix. The cell esds are taken into account individually in the estimation of esds in distances, angles and torsion angles; correlations between esds in cell parameters are only used when they are defined by crystal symmetry. An approximate (isotropic) treatment of cell esds is used for estimating esds involving l.s. planes.
Refinement. Refinement of F^2^ against ALL reflections. The weighted R-factor wR and goodness of fit S are based on F^2^, conventional R-factors R are based on F, with F set to zero for negative F^2^. The threshold expression of F^2^ > 2sigma(F^2^) is used only for calculating R-factors(gt) etc. and is not relevant to the choice of reflections for refinement. R-factors based on F^2^ are statistically about twice as large as those based on F, and R- factors based on ALL data will be even larger.

### Fractional atomic coordinates and isotropic or equivalent isotropic displacement parameters (Å^2^)

**Table:**

	*x*	*y*	*z*	*U*_iso_*/*U*_eq_	
S1	0.99971 (6)	0.92533 (10)	0.81233 (2)	0.02622 (19)	
O1	0.95294 (14)	0.9314 (3)	0.66466 (5)	0.0240 (4)	
O2	1.15707 (16)	0.9326 (3)	0.66746 (5)	0.0376 (5)	
N1	0.93214 (17)	0.9251 (3)	0.72760 (6)	0.0190 (5)	
C1	0.7483 (2)	0.9271 (3)	0.67941 (7)	0.0196 (6)	
C2	0.6604 (2)	0.8757 (3)	0.70390 (7)	0.0189 (6)	
C3	0.6894 (2)	0.8215 (3)	0.74272 (7)	0.0203 (6)	
H3	0.7697	0.8241	0.7541	0.024*	
C4	0.6018 (2)	0.7657 (4)	0.76358 (8)	0.0238 (6)	
H4	0.6232	0.7300	0.7890	0.029*	
C5	0.4792 (2)	0.7611 (4)	0.74733 (8)	0.0250 (6)	
H5	0.4203	0.7209	0.7618	0.030*	
C6	0.4478 (2)	0.8160 (4)	0.71037 (8)	0.0252 (6)	
H6	0.3666	0.8148	0.6998	0.030*	
C7	0.5358 (2)	0.8747 (4)	0.68781 (8)	0.0222 (6)	
C8	0.5026 (2)	0.9324 (4)	0.64940 (8)	0.0259 (6)	
H8	0.4211	0.9341	0.6391	0.031*	
C9	0.5870 (2)	0.9852 (4)	0.62716 (8)	0.0260 (7)	
H9	0.5632	1.0256	0.6022	0.031*	
C10	0.7102 (2)	0.9788 (4)	0.64199 (8)	0.0234 (6)	
H10	0.7675	1.0102	0.6262	0.028*	
C11	0.8774 (2)	0.9272 (3)	0.69294 (7)	0.0200 (6)	
C12	1.0706 (2)	0.9308 (4)	0.68424 (8)	0.0255 (6)	
C13	1.0557 (2)	0.9265 (4)	0.72456 (7)	0.0205 (6)	
C14	1.1464 (2)	0.9264 (4)	0.75406 (8)	0.0217 (6)	
H14	1.2267 (16)	0.931 (4)	0.7486 (7)	0.031 (8)*	
C15	1.1363 (2)	0.9271 (4)	0.79395 (7)	0.0202 (6)	
C16	1.2332 (2)	0.9287 (4)	0.82323 (7)	0.0249 (6)	
H16	1.3139	0.9308	0.8193	0.030*	
C17	1.1949 (2)	0.9268 (4)	0.85972 (8)	0.0285 (7)	
H17	1.2478	0.9270	0.8825	0.034*	
C18	1.0734 (2)	0.9248 (4)	0.85800 (8)	0.0272 (7)	
H18	1.0337	0.9233	0.8796	0.033*	
S1'	0.77988 (6)	0.09732 (10)	0.902561 (19)	0.02376 (18)	
O1'	0.83801 (14)	0.2656 (2)	1.04723 (5)	0.0204 (4)	
O2'	0.63925 (15)	0.2045 (3)	1.04855 (5)	0.0264 (5)	
N1'	0.85670 (17)	0.2013 (3)	0.98541 (6)	0.0169 (5)	
C1'	1.0382 (2)	0.3041 (3)	1.02992 (7)	0.0175 (6)	
C2'	1.1273 (2)	0.2914 (3)	1.00435 (7)	0.0155 (5)	
C3'	1.1032 (2)	0.2400 (4)	0.96522 (7)	0.0195 (6)	
H3'	1.0246	0.2082	0.9547	0.023*	
C4'	1.1931 (2)	0.2364 (4)	0.94265 (8)	0.0220 (6)	
H4'	1.1744	0.2048	0.9169	0.026*	
C5'	1.3133 (2)	0.2796 (4)	0.95750 (8)	0.0224 (6)	
H5'	1.3739	0.2740	0.9419	0.027*	
C6'	1.3403 (2)	0.3296 (4)	0.99488 (8)	0.0226 (6)	
H6'	1.4200	0.3575	1.0047	0.027*	
C7'	1.2499 (2)	0.3402 (3)	1.01921 (7)	0.0175 (6)	
C8'	1.2781 (2)	0.3959 (3)	1.05784 (7)	0.0216 (6)	
H8'	1.3580	0.4243	1.0675	0.026*	
C9'	1.1912 (2)	0.4091 (3)	1.08129 (7)	0.0218 (6)	
H9'	1.2114	0.4485	1.1065	0.026*	
C10'	1.0716 (2)	0.3634 (3)	1.06731 (7)	0.0208 (6)	
H10'	1.0128	0.3730	1.0835	0.025*	
C11'	0.9113 (2)	0.2551 (3)	1.01834 (7)	0.0177 (6)	
C12'	0.7230 (2)	0.2106 (3)	1.03059 (7)	0.0197 (6)	
C13'	0.7361 (2)	0.1687 (3)	0.99081 (7)	0.0175 (6)	
C14'	0.6466 (2)	0.1050 (3)	0.96414 (8)	0.0182 (6)	
H14'	0.5738 (16)	0.084 (3)	0.9725 (6)	0.019 (7)*	
C15'	0.6534 (2)	0.0608 (3)	0.92518 (7)	0.0181 (6)	
C16'	0.5598 (2)	−0.0199 (4)	0.89970 (7)	0.0200 (6)	
H16'	0.4840	−0.0495	0.9062	0.024*	
C17'	0.5937 (2)	−0.0509 (4)	0.86334 (8)	0.0256 (6)	
H17'	0.5430	−0.1050	0.8431	0.031*	
C18'	0.7089 (2)	0.0068 (4)	0.86086 (8)	0.0256 (6)	
H18'	0.7448	−0.0024	0.8386	0.031*	

### Atomic displacement parameters (Å^2^)

**Table:**

	*U*^11^	*U*^22^	*U*^33^	*U*^12^	*U*^13^	*U*^23^
S1	0.0254 (4)	0.0327 (4)	0.0211 (4)	−0.0023 (3)	0.0051 (3)	−0.0003 (3)
O1	0.0216 (9)	0.0343 (11)	0.0166 (10)	0.0025 (8)	0.0043 (8)	0.0011 (9)
O2	0.0263 (10)	0.0643 (15)	0.0240 (12)	0.0058 (10)	0.0098 (9)	0.0037 (11)
N1	0.0182 (11)	0.0220 (12)	0.0169 (12)	0.0003 (10)	0.0023 (9)	0.0003 (10)
C1	0.0244 (13)	0.0154 (13)	0.0189 (14)	0.0006 (11)	0.0026 (11)	−0.0023 (12)
C2	0.0212 (13)	0.0147 (13)	0.0200 (15)	0.0007 (11)	−0.0003 (11)	−0.0037 (11)
C3	0.0203 (13)	0.0204 (14)	0.0201 (15)	0.0013 (12)	0.0020 (11)	−0.0012 (12)
C4	0.0264 (15)	0.0210 (15)	0.0239 (16)	0.0002 (12)	0.0028 (12)	0.0013 (12)
C5	0.0236 (14)	0.0215 (15)	0.0312 (18)	0.0002 (12)	0.0083 (12)	−0.0008 (13)
C6	0.0188 (13)	0.0233 (15)	0.0329 (17)	−0.0008 (12)	0.0013 (12)	−0.0044 (13)
C7	0.0253 (14)	0.0173 (14)	0.0228 (15)	0.0019 (12)	−0.0018 (12)	−0.0036 (12)
C8	0.0221 (14)	0.0262 (16)	0.0270 (16)	0.0045 (12)	−0.0050 (12)	−0.0037 (13)
C9	0.0318 (16)	0.0273 (16)	0.0166 (15)	0.0045 (13)	−0.0046 (12)	0.0015 (12)
C10	0.0273 (15)	0.0232 (15)	0.0198 (15)	0.0016 (12)	0.0034 (12)	−0.0027 (12)
C11	0.0232 (13)	0.0160 (14)	0.0214 (15)	0.0019 (12)	0.0056 (11)	0.0013 (12)
C12	0.0232 (14)	0.0280 (16)	0.0251 (16)	0.0020 (13)	0.0026 (12)	0.0011 (13)
C13	0.0199 (13)	0.0211 (14)	0.0213 (15)	0.0011 (12)	0.0053 (11)	0.0016 (12)
C14	0.0203 (14)	0.0205 (15)	0.0249 (16)	−0.0007 (12)	0.0051 (12)	0.0010 (12)
C15	0.0200 (13)	0.0196 (14)	0.0215 (15)	−0.0013 (12)	0.0044 (11)	0.0009 (12)
C16	0.0332 (15)	0.0194 (14)	0.0216 (16)	0.0021 (13)	0.0019 (12)	−0.0039 (13)
C17	0.0339 (16)	0.0276 (16)	0.0214 (16)	0.0029 (13)	−0.0055 (12)	−0.0013 (13)
C18	0.0335 (16)	0.0315 (17)	0.0169 (15)	−0.0015 (14)	0.0044 (12)	0.0012 (13)
S1'	0.0223 (3)	0.0278 (4)	0.0218 (4)	−0.0035 (3)	0.0053 (3)	−0.0007 (3)
O1'	0.0207 (9)	0.0254 (10)	0.0156 (10)	−0.0007 (8)	0.0042 (7)	−0.0004 (8)
O2'	0.0241 (10)	0.0336 (12)	0.0228 (11)	0.0001 (9)	0.0085 (8)	0.0013 (9)
N1'	0.0168 (10)	0.0155 (11)	0.0184 (12)	0.0010 (9)	0.0028 (9)	0.0007 (9)
C1'	0.0202 (13)	0.0121 (13)	0.0204 (14)	0.0011 (11)	0.0035 (11)	0.0004 (11)
C2'	0.0180 (12)	0.0099 (12)	0.0185 (14)	0.0037 (10)	0.0022 (10)	0.0016 (11)
C3'	0.0206 (13)	0.0193 (14)	0.0184 (15)	−0.0004 (11)	0.0018 (11)	−0.0013 (11)
C4'	0.0275 (15)	0.0215 (15)	0.0173 (14)	0.0012 (12)	0.0039 (11)	−0.0008 (12)
C5'	0.0213 (14)	0.0225 (15)	0.0254 (16)	0.0013 (12)	0.0101 (12)	0.0023 (12)
C6'	0.0193 (13)	0.0189 (14)	0.0300 (17)	0.0012 (12)	0.0052 (12)	0.0029 (13)
C7'	0.0189 (13)	0.0108 (13)	0.0221 (15)	0.0009 (11)	0.0005 (11)	0.0037 (11)
C8'	0.0220 (13)	0.0180 (14)	0.0231 (15)	−0.0022 (11)	−0.0031 (11)	0.0021 (12)
C9'	0.0288 (14)	0.0189 (14)	0.0162 (14)	0.0007 (12)	−0.0018 (11)	−0.0020 (12)
C10'	0.0255 (14)	0.0177 (14)	0.0201 (15)	0.0011 (12)	0.0066 (11)	0.0010 (12)
C11'	0.0249 (14)	0.0144 (13)	0.0149 (14)	0.0036 (11)	0.0069 (11)	0.0017 (11)
C12'	0.0186 (13)	0.0172 (14)	0.0232 (15)	0.0024 (12)	0.0030 (11)	0.0037 (12)
C13'	0.0183 (13)	0.0166 (13)	0.0181 (14)	0.0048 (11)	0.0040 (10)	0.0023 (11)
C14'	0.0133 (12)	0.0191 (14)	0.0231 (15)	0.0015 (11)	0.0054 (11)	0.0034 (12)
C15'	0.0174 (12)	0.0173 (13)	0.0200 (15)	0.0018 (11)	0.0036 (11)	0.0053 (12)
C16'	0.0192 (13)	0.0203 (14)	0.0207 (15)	0.0021 (11)	0.0040 (11)	0.0021 (12)
C17'	0.0204 (14)	0.0294 (16)	0.0251 (16)	−0.0003 (12)	−0.0038 (11)	−0.0015 (13)
C18'	0.0292 (15)	0.0331 (16)	0.0149 (15)	0.0064 (13)	0.0048 (12)	0.0021 (12)

### Geometric parameters (Å, °)

**Table:**

S1—C15	1.735 (3)	S1'—C15'	1.732 (2)
S1—C18	1.704 (3)	S1'—C18'	1.697 (3)
O1—C11	1.391 (3)	O1'—C11'	1.393 (3)
O1—C12	1.396 (3)	O1'—C12'	1.391 (3)
O2—C12	1.199 (3)	O2'—C12'	1.199 (3)
N1—C11	1.289 (3)	N1'—C11'	1.292 (3)
N1—C13	1.397 (3)	N1'—C13'	1.403 (3)
C1—C10	1.379 (3)	C1'—C10'	1.385 (3)
C1—C11	1.453 (3)	C2'—C1'	1.434 (3)
C2—C1	1.440 (3)	C3'—C2'	1.417 (3)
C2—C3	1.415 (3)	C3'—C4'	1.363 (3)
C2—C7	1.427 (3)	C3'—H3'	0.9300
C3—H3	0.9300	C4'—H4'	0.9300
C4—C3	1.361 (3)	C5'—C4'	1.404 (3)
C4—H4	0.9300	C5'—C6'	1.358 (3)
C5—C4	1.407 (3)	C5'—H5'	0.9300
C5—C6	1.359 (4)	C6'—H6'	0.9300
C5—H5	0.9300	C7'—C2'	1.437 (3)
C6—C7	1.408 (4)	C7'—C6'	1.412 (3)
C6—H6	0.9300	C8'—C7'	1.412 (3)
C8—C7	1.414 (4)	C8'—C9'	1.361 (3)
C8—C9	1.358 (4)	C8'—H8'	0.9300
C8—H8	0.9300	C9'—C10'	1.394 (3)
C9—C10	1.401 (3)	C9'—H9'	0.9300
C9—H9	0.9300	C10'—H10'	0.9300
C10—H10	0.9300	C11'—C1'	1.459 (3)
C13—C12	1.454 (4)	C13'—C14'	1.351 (3)
C14—C13	1.347 (3)	C13'—C12'	1.460 (3)
C14—H14	0.942 (16)	C14'—H14'	0.913 (16)
C15—C14	1.426 (4)	C15'—C14'	1.421 (3)
C15—C16	1.388 (3)	C15'—C16'	1.402 (3)
C16—H16	0.9300	C16'—C17'	1.403 (4)
C17—C16	1.410 (4)	C16'—H16'	0.9300
C17—H17	0.9300	C17'—H17'	0.9300
C18—C17	1.348 (3)	C18'—C17'	1.362 (3)
C18—H18	0.9300	C18'—H18'	0.9300
			
C18—S1—C15	91.12 (13)	C18'—S1'—C15'	91.74 (13)
C11—O1—C12	105.42 (19)	C12'—O1'—C11'	106.03 (19)
C11—N1—C13	105.7 (2)	C11'—N1'—C13'	105.6 (2)
C2—C1—C11	122.0 (2)	C2'—C1'—C11'	122.6 (2)
C10—C1—C2	119.7 (2)	C10'—C1'—C2'	119.9 (2)
C10—C1—C11	118.4 (2)	C10'—C1'—C11'	117.5 (2)
C3—C2—C1	124.4 (2)	C1'—C2'—C7'	117.6 (2)
C3—C2—C7	117.6 (2)	C3'—C2'—C1'	125.0 (2)
C7—C2—C1	118.0 (2)	C3'—C2'—C7'	117.4 (2)
C2—C3—H3	119.5	C2'—C3'—H3'	119.4
C4—C3—C2	121.1 (2)	C4'—C3'—C2'	121.1 (2)
C4—C3—H3	119.5	C4'—C3'—H3'	119.4
C3—C4—C5	121.1 (3)	C3'—C4'—C5'	121.3 (2)
C3—C4—H4	119.5	C3'—C4'—H4'	119.4
C5—C4—H4	119.5	C5'—C4'—H4'	119.4
C4—C5—H5	120.3	C4'—C5'—H5'	120.3
C6—C5—C4	119.5 (3)	C6'—C5'—C4'	119.4 (2)
C6—C5—H5	120.3	C6'—C5'—H5'	120.3
C5—C6—C7	121.2 (2)	C5'—C6'—C7'	121.5 (2)
C5—C6—H6	119.4	C5'—C6'—H6'	119.3
C7—C6—H6	119.4	C7'—C6'—H6'	119.3
C6—C7—C2	119.5 (2)	C6'—C7'—C2'	119.2 (2)
C6—C7—C8	121.0 (2)	C8'—C7'—C2'	119.6 (2)
C8—C7—C2	119.4 (2)	C8'—C7'—C6'	121.2 (2)
C7—C8—H8	119.3	C7'—C8'—H8'	119.3
C9—C8—C7	121.5 (2)	C9'—C8'—C7'	121.5 (2)
C9—C8—H8	119.3	C9'—C8'—H8'	119.3
C8—C9—C10	119.8 (3)	C8'—C9'—C10'	119.7 (2)
C8—C9—H9	120.1	C8'—C9'—H9'	120.1
C10—C9—H9	120.1	C10'—C9'—H9'	120.1
C1—C10—C9	121.5 (3)	C1'—C10'—C9'	121.7 (2)
C1—C10—H10	119.2	C1'—C10'—H10'	119.1
C9—C10—H10	119.2	C9'—C10'—H10'	119.1
O1—C11—C1	115.8 (2)	O1'—C11'—C1'	115.2 (2)
N1—C11—O1	115.2 (2)	N1'—C11'—O1'	114.9 (2)
N1—C11—C1	129.0 (2)	N1'—C11'—C1'	129.8 (2)
O1—C12—C13	104.9 (2)	O1'—C12'—C13'	104.7 (2)
O2—C12—O1	121.4 (2)	O2'—C12'—O1'	121.8 (2)
O2—C12—C13	133.7 (2)	O2'—C12'—C13'	133.5 (2)
N1—C13—C12	108.8 (2)	N1'—C13'—C12'	108.8 (2)
C14—C13—N1	125.8 (2)	C14'—C13'—N1'	126.2 (2)
C14—C13—C12	125.5 (2)	C14'—C13'—C12'	125.0 (2)
C13—C14—C15	127.5 (2)	C13'—C14'—C15'	127.8 (2)
C13—C14—H14	118.3 (16)	C13'—C14'—H14'	115.7 (15)
C15—C14—H14	114.2 (16)	C15'—C14'—H14'	116.5 (15)
C14—C15—S1	124.13 (19)	C14'—C15'—S1'	124.58 (19)
C16—C15—S1	110.8 (2)	C16'—C15'—S1'	110.32 (19)
C16—C15—C14	125.0 (2)	C16'—C15'—C14'	125.1 (2)
C15—C16—C17	112.1 (2)	C15'—C16'—C17'	112.3 (2)
C15—C16—H16	124.0	C15'—C16'—H16'	123.9
C17—C16—H16	124.0	C17'—C16'—H16'	123.9
C16—C17—H17	123.6	C16'—C17'—H17'	123.6
C18—C17—C16	112.8 (2)	C18'—C17'—C16'	112.8 (2)
C18—C17—H17	123.6	C18'—C17'—H17'	123.6
S1—C18—H18	123.4	S1'—C18'—H18'	123.5
C17—C18—S1	113.1 (2)	C17'—C18'—S1'	112.9 (2)
C17—C18—H18	123.4	C17'—C18'—H18'	123.5
			
C15—S1—C18—C17	0.3 (2)	C18'—S1'—C15'—C14'	179.3 (2)
C18—S1—C15—C14	179.3 (2)	C18'—S1'—C15'—C16'	−0.2 (2)
C18—S1—C15—C16	−0.5 (2)	C15'—S1'—C18'—C17'	−0.3 (2)
C12—O1—C11—N1	0.3 (3)	C12'—O1'—C11'—N1'	0.4 (3)
C12—O1—C11—C1	180.0 (2)	C12'—O1'—C11'—C1'	−178.9 (2)
C11—O1—C12—O2	179.5 (3)	C11'—O1'—C12'—O2'	179.7 (2)
C11—O1—C12—C13	−0.1 (3)	C11'—O1'—C12'—C13'	0.0 (2)
C13—N1—C11—O1	−0.4 (3)	C13'—N1'—C11'—O1'	−0.6 (3)
C13—N1—C11—C1	−180.0 (2)	C13'—N1'—C11'—C1'	178.5 (2)
C11—N1—C13—C12	0.3 (3)	C11'—N1'—C13'—C12'	0.6 (3)
C11—N1—C13—C14	179.5 (3)	C11'—N1'—C13'—C14'	−178.0 (2)
C2—C1—C10—C9	0.8 (4)	C2'—C1'—C10'—C9'	−1.0 (4)
C11—C1—C10—C9	−178.8 (2)	C11'—C1'—C10'—C9'	178.5 (2)
C2—C1—C11—O1	164.0 (2)	C3'—C2'—C1'—C10'	−177.3 (2)
C2—C1—C11—N1	−16.4 (4)	C3'—C2'—C1'—C11'	3.2 (4)
C10—C1—C11—O1	−16.3 (3)	C7'—C2'—C1'—C10'	0.6 (3)
C10—C1—C11—N1	163.3 (3)	C7'—C2'—C1'—C11'	−178.9 (2)
C3—C2—C1—C10	−179.6 (2)	C4'—C3'—C2'—C1'	178.2 (2)
C3—C2—C1—C11	0.1 (4)	C4'—C3'—C2'—C7'	0.2 (4)
C7—C2—C1—C10	2.0 (4)	C2'—C3'—C4'—C5'	1.4 (4)
C7—C2—C1—C11	−178.3 (2)	C6'—C5'—C4'—C3'	−1.3 (4)
C1—C2—C3—C4	−176.7 (2)	C4'—C5'—C6'—C7'	−0.3 (4)
C7—C2—C3—C4	1.7 (4)	C6'—C7'—C2'—C1'	−179.9 (2)
C1—C2—C7—C6	176.8 (2)	C6'—C7'—C2'—C3'	−1.8 (3)
C1—C2—C7—C8	−3.1 (4)	C8'—C7'—C2'—C1'	0.6 (3)
C3—C2—C7—C6	−1.7 (4)	C8'—C7'—C2'—C3'	178.8 (2)
C3—C2—C7—C8	178.4 (2)	C2'—C7'—C6'—C5'	1.9 (4)
C5—C4—C3—C2	−0.4 (4)	C8'—C7'—C6'—C5'	−178.7 (2)
C6—C5—C4—C3	−1.0 (4)	C9'—C8'—C7'—C2'	−1.6 (4)
C4—C5—C6—C7	1.0 (4)	C9'—C8'—C7'—C6'	179.0 (2)
C5—C6—C7—C2	0.3 (4)	C7'—C8'—C9'—C10'	1.2 (4)
C5—C6—C7—C8	−179.8 (3)	C8'—C9'—C10'—C1'	0.1 (4)
C9—C8—C7—C2	1.4 (4)	O1'—C11'—C1'—C2'	176.7 (2)
C9—C8—C7—C6	−178.5 (3)	O1'—C11'—C1'—C10'	−2.8 (3)
C7—C8—C9—C10	1.5 (4)	N1'—C11'—C1'—C2'	−2.4 (4)
C8—C9—C10—C1	−2.7 (4)	N1'—C11'—C1'—C10'	178.0 (2)
N1—C13—C12—O1	−0.1 (3)	N1'—C13'—C12'—O1'	−0.4 (3)
N1—C13—C12—O2	−179.6 (3)	N1'—C13'—C12'—O2'	180.0 (3)
C14—C13—C12—O1	−179.3 (3)	C14'—C13'—C12'—O1'	178.3 (2)
C14—C13—C12—O2	1.1 (5)	C14'—C13'—C12'—O2'	−1.4 (5)
C15—C14—C13—N1	−0.9 (5)	N1'—C13'—C14'—C15'	−1.2 (4)
C15—C14—C13—C12	178.3 (3)	C12'—C13'—C14'—C15'	−179.7 (2)
S1—C15—C14—C13	0.9 (4)	S1'—C15'—C14'—C13'	−5.1 (4)
C16—C15—C14—C13	−179.4 (3)	C16'—C15'—C14'—C13'	174.3 (3)
S1—C15—C16—C17	0.5 (3)	S1'—C15'—C16'—C17'	0.7 (3)
C14—C15—C16—C17	−179.3 (3)	C14'—C15'—C16'—C17'	−178.8 (2)
C18—C17—C16—C15	−0.3 (3)	C15'—C16'—C17'—C18'	−0.9 (3)
S1—C18—C17—C16	−0.1 (3)	S1'—C18'—C17'—C16'	0.8 (3)

### Hydrogen-bond geometry (Å, °)

**Table:**

*D*—H···*A*	*D*—H	H···*A*	*D*···*A*	*D*—H···*A*
C3—H3···N1	0.93	2.27	2.924 (3)	127
C3'—H3'···N1'	0.93	2.29	2.946 (3)	127
C6—H6···O2^i^	0.93	2.59	3.483 (3)	160
C9'—H9'···O2^ii^	0.93	2.46	3.310 (3)	152
C16'—H16'···O2'^iii^	0.93	2.50	3.329 (3)	149

Symmetry codes: (i) *x*−1, *y*, *z*; (ii) *x*, −*y*+3/2, *z*+1/2; (iii) −*x*+1, −*y*, −*z*+2.
